# Glycyrrhizin Protects the Diabetic Retina against Permeability, Neuronal, and Vascular Damage through Anti-Inflammatory Mechanisms

**DOI:** 10.3390/jcm8070957

**Published:** 2019-07-02

**Authors:** Li Liu, Youde Jiang, Jena J. Steinle

**Affiliations:** Visual, and Anatomical Sciences, Department of Ophthalmology, Wayne State University, Detroit, MI 48202, USA

**Keywords:** diabetic retinopathy, inflammation, endothelial cells, glycyrrhizin, high mobility group box 1 (HMGB1)

## Abstract

Damage associated molecular pattern (DAMPs), such as high mobility group box 1 (HMGB1), may be involved in retinal inflammation in response to high glucose. To test whether HMGB1 inhibition could protect the diabetic retina, C57BL/6J mice were made diabetic and treated with glycyrrhizin, a HMGB1 inhibitor, for up to six months. Measurements of permeability, neuronal, and vascular changes were done, as well as assessments of HMGB1, tumor necrosis factor alpha (TNFα), and interleukin-1-beta (IL1β) levels. Retinal endothelial cells (REC) treated with glycyrrhizin had reduced IL1β and cleaved caspase 3 levels. Data also demonstrate that glycyrrhizin effectively reduced HMGB1 levels throughout the retina, as well as maintained normal retinal permeability and retinal capillary coverage. Glycyrrhizin maintained normal cell numbers in the ganglion cell layer and prevented thinning of the retina at two months. These histological changes were associated with reduced reactive oxygen species, as well as reduced HMGB1, TNFα, and IL1β levels. The data strongly imply that HMGB1 inhibition prevented diabetic retinal changes through anti-inflammatory pathways.

## 1. Introduction

Despite ongoing attempts to prevent and/or delay progression of diabetic retinopathy, it remains the leading cause of blindness in younger adults. Anti-vascular endothelial growth factor (VEGF) therapy is effective for some patients with macular edema or proliferative disease. However, little is available for early stage disease.

For the past two decades, diabetes and diabetic retinopathy have become increasingly linked to chronic inflammation (Joussen et al., 2004 [1], Tang and Kern 2011 [2]). Recent discoveries have shown associations between retinal pathology and a large number of inflammatory mediators (tumor necrosis factor alpha (TNFα), interleukin-1-beta (IL1β), inducible nitric oxide synthase inducible nitric oxide synthase (iNOS), Fas ligands) (Joussen et al., 2009 [3], Vincent and Mohr 2007 [4], Romeo et al., 2002 [5], Du, Sarthy, and Kern 2004 [6]) and innate immune components (Toll-like receptor 2 and 4 (TLR2 and TLR4)) (Wang et al., 2015 [7], Rajamani and Jialal 2014 [8], Berger et al., 2016 [9], Tang and Kern 2011 [2]). In addition, some have suggested that diabetes may represent a form of sterile inflammation, leading to activation of high mobility group box 1 (HMGB1), a damage associated molecular pattern (DAMP) (Tsung, Tohme, and Billiar 2014 [10], Andersson and Tracey 2011 [11]).

We chose to focus on HMGB1, as research in one-month diabetic rats showed that glycyrrhizin, a HMGB1 inhibitor, significantly reduced HMGB1, extracellular signal-related protein kinases 1 and 2 (ERK1/2), caspase-3, and glutamate levels (Abu El-Asrar et al., 2014 [12]). Additionally, studies on receptor for advanced glycation end products (RAGE) knockout mice showed that ischemia/reperfusion (I/R) significantly increased HMGB1 levels in the retina (Dvoriantchikova et al., 2011 [13]). Inhibition of HMGB1 reduced neuronal cell loss in these mice. In other ocular targets, results in a model of *Pseudomonas aeruginosa* keratitis showed that glycyrrhizin significantly reduced HMGB1 levels and bacterial load (Ekanayaka et al., 2016 [14]). Glycyrrhizin is a natural anti-inflammatory and antifungal factor, which inhibits HMGB1 chemoattractant and mitogenic activities through direct binding to HMGB1 and interacting with the two shallow surfaces on the arms of the HMGB1 A and B boxes (Mollica et al., 2007 [15]). Studies using recurrent seizure models showed that inhibition of HMGB1 is protective against epileptic events (Morales-Sosa et al., 2018 [16]). We recently used glycyrrhizin to show that acute inhibition of HMGB1 protected against I/R-induced damage to the retina (Liu, Jiang, and Steinle 2017 [17]). However, no studies have investigated the chronic effects of glycyrrhizin in the diabetic retina. 

We hypothesized that long-term inhibition of HMGB1 using glycyrrhizin would protect the retina against diabetes-induced damage.

## 2. Experimental Section

### 2.1. Mice

Male C57BL/6J mice were purchased from Jackson Laboratories at eight weeks of age. Mouse experiments were approved by the Institutional Animal Care and Use Committee at Wayne State University (Protocol# 17-07-301) and adhere to the Animal Policy of the Association for Research in Vision and Ophthalmology. Diabetes was induced by 60 mg/kg injections of streptozotocin dissolved in citrate buffer for up to five consecutive days. Control mice received citrate buffer only. Glucose measurements were done each week, with glucose levels >250 mg/dL accepted as diabetic. Mice were not fasted before glucose measurements, and all measurements were taken on ~5 µL blood samples measured by a handheld measurement device. Table 1 provides body weights and glucose measurements from all mice. Five mice each were used for permeability, neuronal, and vascular analyses. Five mice at both two and six months were used for protein analyses.

A subset of the control and diabetic mice will then be treated with glycyrrhizin in their drinking water (150 mg/kg/day) (Abu El-Asrar et al., 2014 [12]). Mice were maintained on the drinking water for six months. Water consumption was measured weekly for the first month, and then twice a week to ensure that mice were consuming the correct dose of drug. Based on mouse water consumption, glycyrrhizin did not appear to change the taste of the water. We also measured HMGB1 levels at both two and six months to demonstrate that the dose of glycyrrhizin was effectively reducing HMGB1 levels. 

### 2.2. Measurement of Permeability Changes in the Retina 

Permeability analyses were completed on control and diabetic mice alone and following glycyrrhizin treatment at two and six months. For fluorescein angiography (FA), the pupil was dilated with tropicamide ophthalmic solution, followed by anesthesia via ketamine and xylazine. After mice were deeply anesthetized, 150 µL of AK-FLUOR (1% W/V, AKORN, INC, Lake Forest, IL, USA) was injected intraperitoneally (IP). Retinal vessel leakage was imaged by a Micron IV (Phoenix Research Labs, Pleasanton, CA, USA). Images were taken less than 5 min after injection of the dye. 

For a more quantitative measurement of permeability, additional mice were transfused with 200 µL, Evans blue (0.5% in saline, Sigma Aldrich) via the tail vein. Forty-five minutes after infusion, mice were euthanized with CO2, with retinas were carefully removed, placed into 100 µL formamide, and incubated for 48 h at 55 °C. Tubes were then centrifuged and transferred to a 96-well plate for measurements of the absorbance at measured at 610 (Radu and Chernoff 2013 [18]). 

### 2.3. Measurement of Neuronal Damage

After two months of diabetes or diabetes + glycyrrhizin treatment, a subset of each group of mice was sacrificed for measurements of neuronal thickness, as we have done previously with the exception of staining with hematoxylin and eosin instead of toluidine blue (Steinle et al., 2009 [19]). Analyses of retinal thickness and cell numbers for each retinal layer were assessed from the same regions in each retina, as we have done in the past (Zhang et al., 2012 [20], Steinle et al., 2009 [19]).

### 2.4. Measurement of Degenerate Capillaries 

After six months of diabetes or treatment, the remaining mice were sacrificed. Some mice were processed for measurements of degeneration of capillaries as we have done in the past (Liu, Jiang, and Steinle 2016 [21], Veenstra et al., 2015 [22]).

### 2.5. Measurement of Reactive Oxygen Species

Protein lysates from all groups of mice at both two and six months of diabetes and treatment were processed for measurement of reactive oxygen species (ROS) using the 2’-7’-Dichlorodihydrofluorescein diacetate (DCFDA) method. Briefly, equal protein from each group was loaded into a black 96-well plate and treated with the DCFDA in triplicate and read on a fluorescent plate reader set with an excitation of 485 nm and emission at 530 nm. Some wells were left blank, and some wells only received the DCFDA reagent. The blanks and dye only wells were subtracted from the raw data (Piippo et al., 2018 [23]). Data are plotted as the fluorescence intensity. In addition to the measurement of ROS using the DCFDA method, we also performed Western blotting to measures of 4-HNE to determine whether glycyrrhizin reduced lipid peroxidation in the retina of mice. 

### 2.6. Retinal Endothelial Cells (REC) Cultures 

Primary human retinal endothelial cells (REC) were purchased from Cell Systems Corporation (CSC, Kirkland, Washington, DC, USA). Cells were grown in basal glucose medium supplemented with microvascular growth factors (MVGS), 10 µg/mL gentamycin, and 0.25 µg/mL amphotericin B (Invitrogen, Carlsbad, CA, USA). Once cells reached confluence, some dishes were moved to Cell Systems high glucose medium. Only dishes prior to passage 6 were used. Cells were quiesced by incubating in high or normal glucose medium without MVGS for 24 h prior to experimental use. REC in normal (5 mM) and high glucose (25 mM) were treated with glycyrrhizin (2 mM for 2 h) (Matsui et al., 2004 [24]). 

### 2.7. Western Blotting 

At both two and six months of diabetes and treatment or REC treatment with glycyrrhizin, whole retinal/cell lysates were collected into lysis buffer containing protease and phosphatase inhibitors. Equal amounts of protein from the cell extracts were separated on the pre-cast tris-glycine gel (Invitrogen, Carlsbad, CA, USA), blotted onto a nitrocellulose membrane. After blocking in TBST (10 mM Tris-HCl buffer, pH 8.0, 150 mM NaCl, 0.1% Tween 20) and 5% (*w/v*) bovine serum albumin (BSA), the membranes were treated with an HMGB1 (1:500, ab227168) or 4-HNE (1:500, ab46545) (Abcam, Cambridge, MA, USA) or beta actin-HRP (1:2000, sc-47778, Santa Cruz Biotechnology, Santa Cruz, CA, USA) primary antibodies. Antigen-antibody complexes were detected by chemiluminescence reagent kit (Thermo Scientific, Pittsburgh, PA, USA). Data was acquired using an Azure C500 (Azure Biosystems, Dublin, CA, USA). Western blot analyses were done using Image Studio Light software.

### 2.8. Enzyme-Linked Immosorbent Assay

A TNFα enzyme-linked immunosorbent assay (ELISA) (Fisher Scientific, Pittsburgh, PA, USA) was completed according to manufacturer’s instructions with the exception that samples were exposed to primary antibody for 24 h, and 100 µg of protein was used to insure equal protein amounts in all wells. The IL1β ELISA was completed according to manufacturer’s instructions with the exception that 120 µg protein loaded into all wells, and the primary antibody was incubated overnight. A cleaved caspase 3 ELISA was done following manufacturer’s instructions (Cell Signaling, Danvers, MA, USA), with equal protein loaded to allow for measurements using optical density (O.D.) values. 

### 2.9. Statistics 

A one-way ANOVA with Student Newman Keul’s post-hoc test was used for data analyses on animal samples. A Kruskal-Wallis analysis was done for cell culture data. All analyses were done using Prism software by GraphPad (San Diego, CA, USA). Some data numbers may not reach the 80% level by power analyses; however, the numbers used for these studies are standard for the field (Abcouwer et al., 2010 [25], Al-Shabrawey et al., 2008 [26]). Data are mean ± standard error of mean unless stated otherwise. *p* < 0.05 was considered statistically significant. For all data, *n* = the number of mice or cells per treatment group. A representative blot is given for Western blot data. 

## 3. Results

### 3.1. Glycyrrhizin Reduced Blood Glucose Levels at Two Months, but Not Six Months 

Table 1 data demonstrates that type 1 diabetic mice treated with glycyrrhizin for two months had a reduction in blood glucose levels compared to diabetic mice receiving no treatment (*p* < 0.05, 1-way ANOVA analyses). This was not maintained at six months. Our findings are different from published data at one month of glycyrrhizin, which found no differences in blood glucose at one month of diabetes and glycyrrhizin treatment in rats (Abu El-Asrar et al., 2014 [12]). Since we used the same dosing for glycyrrhizin, the reason for these differences is unclear. Our data do agree with other diabetic models, including periodontal disease (Akutagawa et al., 2019 [27]) and kidney disease (Wang et al., 2014 [28]). Nonetheless, in our studies, glycyrrhizin only had a short-term effect on blood glucose in these mice, so the chronic actions of glycyrrhizin on the retina likely result from other mechanisms. 

### 3.2. Glycyrrhizin Reduced Diabetes-Induced Permeability at Both Two and Six Months 

**Figure 1:**
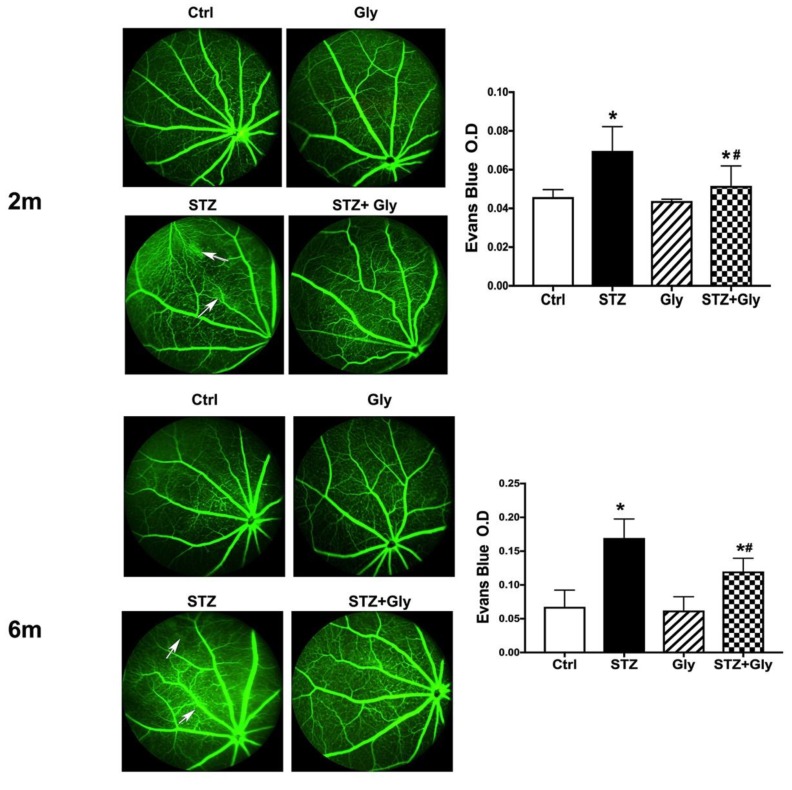
Fluorescein angiography (left) and Evan’s blue analyses (right) from control mice (Ctrl), control mice treated with glycyrrhizin (Gly), diabetic mice (STZ), and diabetic mice treated with glycyrrhizin (STZ + Gly) at two months (top) and six months (bottom). Arrows point to areas of leakage. *p < 0.05 vs. ctrl, #*p* < 0.05 vs. Diabetes. *n* = 5 for each group.

To investigate changes in retinal permeability due to diabetes, we performed fluorescein angiography and Evan’s blue measurements at two months and six months of diabetes in all groups. Figure 1A shows the angiography results at two months. The data demonstrate increased leakage in the type 1 diabetic group, which is reduced with glycyrrhizin treatment. This is quantified in Figure 1B using Evan’s blue (*p < 0.05 via one-way ANOVA). At six months, we see the same pattern of increased leakage due to diabetes (Figure 1C), which is reduced by glycyrrhizin treatment, both in angiography and using Evan’s blue methods (Figure 1D, *p* < 0.05). Glycyrrhizin had limited effects on permeability in the control retina.

### 3.3. Neuronal Measurements Were Increased in Mice Receiving Glycyrrhizin Compared to Diabetic Only Retina at Two Months 

**Figure 2:**
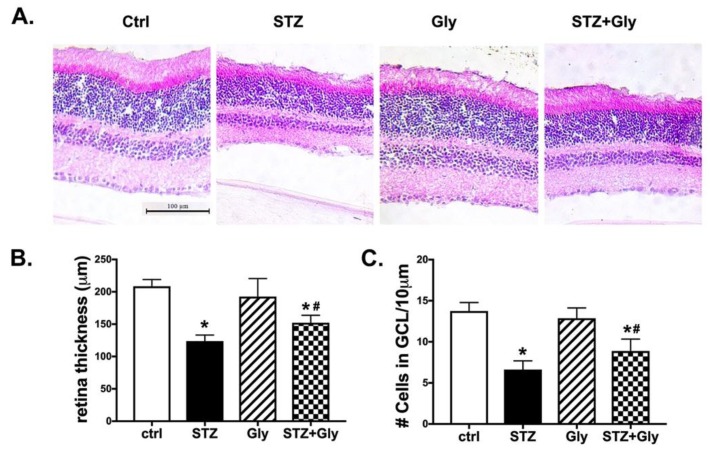
Hematoxylin and eosin for neuronal changes at two months of treatment. (**A**) Are representative images from control mice (Ctrl), control mice treated with glycyrrhizin (Gly), diabetic mice (STZ), and diabetic mice treated with glycyrrhizin (STZ + Gly). (**B**) Shows quantification of retinal thickness, and (**C**) is data on cell numbers in the ganglion cell layer (GCL). * *p* < 0.05 vs. ctrl, # *p*< 0.05 vs. Diabetes. *n* = 5 for each group.

To investigate whether glycyrrhizin protected the retina against neuronal damage, we measured retinal thickness and cell numbers in the ganglion cell layer after two months of diabetes or diabetes and glycyrrhizin treatment, and analyzed data using a one-way ANOVA. Figure 2 shows that the type 1 diabetic retina is much thinner than control mice, control mice treated with glycyrrhizin, or diabetic mice treated with glycyrrhizin. Figure 2B is quantitation of retinal thickness showing that glycyrrhizin significantly increased retinal thickness in diabetic mice (*p* < 0.05). Figure 2C shows that glycyrrhizin treatment also increased the cell numbers in the ganglion cell layer compared to diabetic mice (*p* < 0.05). Glycyrrhizin alone did not alter neuronal measurements. 

### 3.4. Glycyrrhizin Protected the Retinal Vasculature against Diabetes-Induced Injury at Six Months 

**Figure 3:**
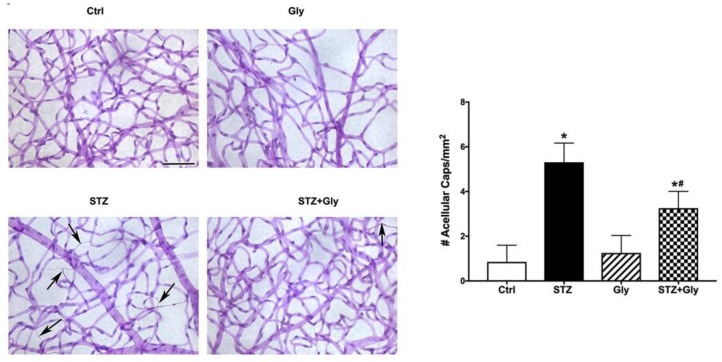
Vascular changes in the diabetic retina. Measurement of degenerate capillaries from control mice (Ctrl), control mice treated with glycyrrhizin (Gly), diabetic mice (STZ), and diabetic mice treated with glycyrrhizin (STZ + Gly). Representative images are presented on the left, with quantification on the right. Arrows point to degenerate capillaries. * *p* < 0.05 vs. ctrl, # *p* < 0.05 vs. diabetes. *n* = 5 for each group. Scale bar is 50 µm.

Diabetes causes the formation of degenerate capillaries after six months of exposure to high glucose (Zheng et al., 2007 [29], Zhang et al., 2012 [20]). We wanted to ascertain if glycyrrhizin could reduce retinal vascular damage. Figure 3 shows that diabetes significantly increased the numbers of degenerate capillaries, which was reduced by glycyrrhizin treatment (*p* < 0.05). Data were analyzed using a one-way ANOVA. Control mice treated with glycyrrhizin had no response to treatment. 

### 3.5. Glycyrrhizin Significantly Reduced Reactive Oxygen Species Levels in Retina Lysates at Both Two and Six Months 

**Figure 4:**
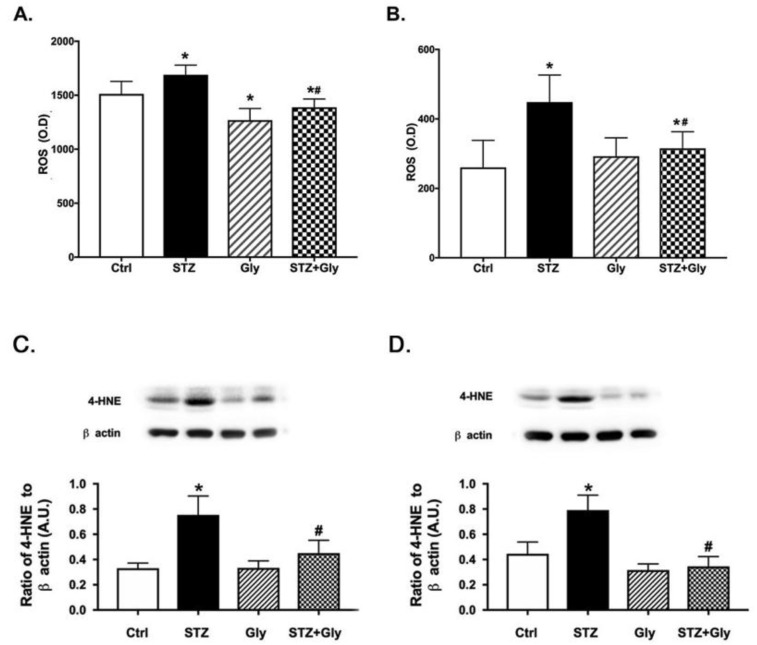
Reactive oxygen species (ROS) measurement in diabetic mice. (**A**) is at two months and (**B**) is measurement of ROS in control mice (Ctrl), control mice treated with glycyrrhizin (Gly), diabetic mice (STZ), and diabetic mice treated with glycyrrhizin (STZ + Gly). (**C**,**D**) show 4-HNE levels in the same groups. * *p* < 0.05 vs. ctrl, # *p* < 0.05 vs. diabetes. *n* = 5 for each group.

Retinal damage from diabetes is reported to be caused by a significant increase in ROS production (Tang and Kern 2011 [2], Brownlee 2001 [30]). Therefore, we investigated whether glycyrrhizin could reduce ROS production in whole retinal lysates. Figure 4A,B shows that glycyrrhizin significantly reduced ROS levels in the diabetic retina, with limited effects in the control retina based on analyses using a one-way ANOVA. It is noted that total ROS levels were reduced at six months vs. two months in these mice. However, the rationale for this finding will be explored in the future. To further confirm the actions of glycyrrhizin on the retina, we also measured 4-HNE levels, a marker of lipid peroxidation. Figure 4C,D show that diabetes increased lipid peroxidation in the retina, which was inhibited by glycyrrhizin treatment (*p* < 0.05).

### 3.6. Diabetes Increased HMGB1 Levels, TNFα, and IL1β Levels in the Retina, Which Was Reduced Following Glycyrrhizin Treatment 

**Figure 5:**
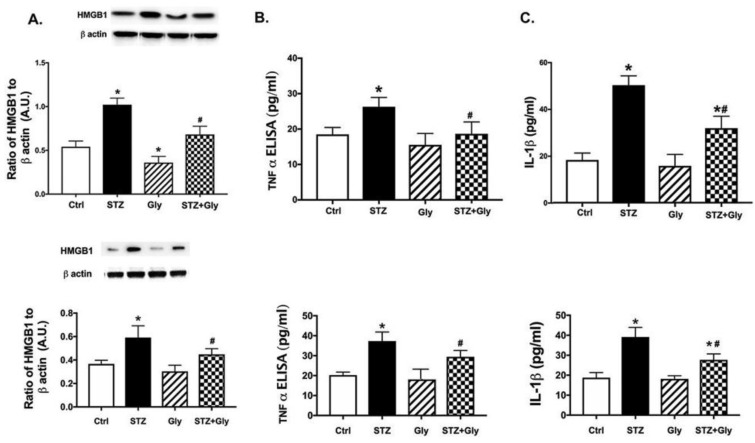
Inflammatory mediators are increased in diabetes. Top panels are at two months and bottom panels are at six months of diabetes. (**A**) represents high mobility group box 1 (HMGB1), (**B**) is tumor necrosis factor alpha (TNFα), and (**C**) is interleukin-1-beta (IL1β) in control mice (Ctrl), control mice treated with glycyrrhizin (Gly), diabetic mice (STZ), and diabetic mice treated with glycyrrhizin (STZ + Gly). * *p* < 0.05 vs. ctrl, # *p* < 0.05 vs. Diabetes. *n* = 5 for each group.

We previously showed that HMGB1 and inflammatory mediators are increased in REC grown in high glucose (Liu, Jiang, and Steinle 2017 [17]). However, we wanted to expand those findings into the diabetic retina. Figure 5 shows that diabetes increased HMGB1 (A), TNFα (B), and IL1β (C) levels in the diabetic retina at both two (top) and six months (bottom), which were significantly reduced by systemic treatment with glycyrrhizin (*p* < 0.05, one-way ANOVA analyses). 

### 3.7. Glycyrrhizin Blocks Both IL1β and Cleaved Caspase 3 in REC Exposed to High Glucose 

**Figure 6:**
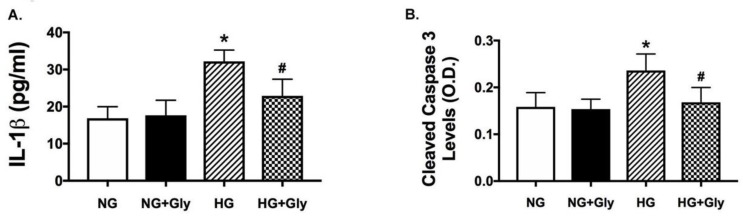
IL1β and cleaved caspase 3 are reduced by glycyrrhizin in retinal endothelial cells (REC) grown in high glucose. REC were grown in normal (5 mM) or high (25 mM) glucose alone or treated with 2mM glycyrrhizin. (**A)** shows IL1β and (**B)** shows cleaved caspase 3. * *p* < 0.05 vs. NG, # *p* < 0.05 vs. HG. *n* = 5 for each group.

Glycyrrhizin reduced TNFα and HMGB1 levels in REC grown in high glucose (Liu, Jiang, and Steinle 2017 [17]), so we decided to further dissect a mechanism for glycyrrhizin by measuring IL1β and cleaved caspase 3 in REC grown in normal and high glucose and treated with glycyrrhizin. Figure 6 demonstrates that REC grown in high glucose medium had increased levels of IL1β and cleaved caspase 3, which agrees with previous work. Similar to the mouse data, glycyrrhizin significantly decreased IL1β levels and cleaved caspase 3 in the REC exposed to high glucose (*p* < 0.05 via Kruskal Wallis testing). Combined with our previous studies in the retinal vasculature, glycyrrhizin likely protects the diabetic retina through anti-inflammatory actions. 

## 4. Discussion

Although a large number of cytokines are involved in diabetic complications, we focused the present study on HMGB1. HMGB1 has been associated with diabetes or high glucose in several retinal cell types, including pericytes (Kim et al. 2016 [31]), endothelial cells (Liu, Jiang, and Steinle 2017 [17]), and glial cells and retinal ganglion cells (Santos et al. 2014 [32], Dvoriantchikova et al., 2011 [13]). Inhibition of HMGB1 could be developed as a therapy for diabetic disease. HMGB1 siRNA improved electroretinogram (ERG) measurements, retinal morphology, and reduced retinal cell apoptosis when given intravitreally to diabetic rats or REC grown in high glucose (Jiang and Chen 2017 [33]). Studies in ARPE-19 cells demonstrated that HMGB1 upregulated angiogenic and fibrogenic factors in response to hypoxia (Chang et al. 2017 [34]). Our data support the findings by El-Asrar (2014) showing reduced apoptotic proteins and loss of neuronal proteins at one month of diabetes in rats (Abu El-Asrar et al., 2014 [12]). Unlike their findings in rats, we found that glycyrrhizin reduced blood glucose at two months, but not six months in mice. When we used glycyrrhizin in the drinking water of diabetic mice over six months, we found that glycyrrhizin improved permeability, neuronal, and vascular damage associated with diabetes through a decrease in HMGB1. We previously reported that I/R-induced neuronal and vascular damage was improved after glycyrrhizin (Liu, Jiang, and Steinle 2017 [17]). Our data support the findings of a role of HMGB1 inhibition on retinal ganglion cells in a model of I/R (Dvoriantchikova et al., 2011 [13]). The results of the present study agree with acute experiments in diabetic rats on neuronal protection (Abu El-Asrar et al., 2014 [12]), and expand existing literature to investigate the chronic, preventative actions of glycyrrhizin on both neuronal and vascular changes in the diabetic retina. 

We also wanted to investigate potential mechanisms by which glycyrrhizin protected the diabetic retina. Glycyrrhizin reduced TNFα in REC grown in high glucose [17]. We extended those findings in the present study to show that glycyrrhizin also reduced IL1β and cleaved caspase 3 in REC grown in high glucose, suggesting that glycyrrhizin protects the diabetic retina through anti-inflammatory actions. We confirmed that glycyrrhizin significantly reduced HMGB1 after systemic treatment at both two and six months of treatment via drinking water. We also show that TNFα and IL1β were significantly reduced by glycyrrhizin, further suggesting that glycyrrhizin works through anti-inflammatory actions. Our findings agrees with studies in the diabetic kidney that showed reduced inflammatory mediators and renal lesions following glycyrrhizin treatment to diabetic rats (Zhang et al., 2017 [35]). In diabetic mice, other authors reported that glycyrrhizin reduced nuclear factor kappa beta (NFkB) and P38 in diabetic kidneys (Wang et al., 2014 [28]). 

In addition to anti-inflammatory actions, we also demonstrated that glycyrrhizin reduced ROS. Diabetes has long been considered a disease resulting from oxidative stress (Brownlee 2001 [30]). Studies on vitreous samples of patients with proliferative diabetic retinopathy demonstrated that the samples had increased HMGB1 levels, as well as markers of oxidative stress (Abu El-Asrar et al., 2017 [36]). The same authors also showed that HMGB1 increased ROS levels in REC (Mohammad et al., 2015 [37]). We found that diabetes significantly increased ROS levels in whole retinal lysates, which was reduced with two and six months of glycyrrhizin treatment. We also found that glycyrrhizin reduced 4-HNE levels in the retinal lysates, suggested reduced lipid peroxidation. Our data agree with findings in the diabetic kidney (Hou et al. 2014 [38]). 

Our data strongly suggest that glycyrrhizin is protective to the diabetic retina when given at the initiation of diabetes. This is not ideal for a chronic disease. Long-term interventional studies on the actions of glycyrrhizin when initiated at six months of diabetes are in their initial phases. Additionally, while our studies confirm previous studies on the acute actions of glycyrrhizin on the diabetic retina, we demonstrate the vascular actions of glycyrrhizin for the first time in the diabetic retina. We also provide data suggesting that glycyrrhizin’s actions are through the inhibition of inflammatory pathways activated by HMBG1, as well as a reduction in ROS. Future studies will further dissect exact mechanisms by which glycyrrhizin can protect the diabetic retina. 

## 5. Conclusions

In conclusion, these data strongly suggest that oral delivery of glycyrrhizin may prevent the complications involved in diabetic retinopathy. While glycyrrhizin is already available for treatment of blepharitis and bone metabolism and used as a treatment for a variety of disorders in Asian cultures (Omar et al., 2012 [39]), use in diabetic retinopathy has not been investigated. Nonetheless, glycyrrhizin offers an additional option for therapy development for retinal pathology in diabetes. 

## Figures and Tables

**Table 1:** Data are mean ± standard deviation.* *p* < 0.05 vs ctrl, ^#^ *p* < 0.05 Vs. Diabetes. Ctrl, control. Diabetes, Gly, glycyrrhizin.

	Ctrl		Ctrl + Gly		Diabetes		Diabetes + Gly	
	BW (g)	BG	BW (g)	BG	BW (g)	BG	BW (g)	BG
8w	25.6 ± 2.2	113 ± 12	25.8 ± 1.9	109 ± 6.7	25.2 ± 2.0	110 ± 8.1	25.3 ± 1.8	108 ± 7.2
2 m Diabetes	32 ± 2	109 ± 8	30 ± 1.9	110 ± 6.8	26 ± 1.4 ^*^	397 ± 119^*^	28 ± 1.2	278 ± 46 ^#*^
6 m Diabetes	35.6 ± 2.6	110 ± 12.6	33.5 ± 2	129 ± 21.2	25.5 ± 2.9 ^*^	538 ± 73.6^*^	26.2 ± 3.5	502 ± 101 ^*^

BW = body weight, BG = blood glucose.
